# Supplementary material for: The Systems Biology Research Tool: evolvable open-source software
Source: BMC Syst Biol. 2008 Jun 29;2:55. doi: 10.1186/1752-0509-2-55 (PMC2446383; doi:10.1186/1752-0509-2-55)
Supplement: Additional file 1 — SBRT Archive. An archive of the current version of the Systems Biology Research Tool. [file 1752-0509-2-55-S1.zip › sbrt-1.4.0/doc/developers_guide/api/sbrt/kernel/fba/comp/CycleComputer.html]

CycleComputer


|  |  |  |
| --- | --- | --- |
| **Overview** \| **Package** \| **Class** \| **Use** \| **Tree** \| **Deprecated** \| **Index** \| **Help** | |  |
| **PREV CLASS**   **NEXT CLASS** | **FRAMES**    **NO FRAMES**     **All Classes** |  |
| SUMMARY: NESTED \| FIELD \| CONSTR \| METHOD | DETAIL: FIELD \| CONSTR \| METHOD |  |


---


## sbrt.kernel.fba.comp Interface CycleComputer

**All Known Implementing Classes:**: SlpCycleComputer

---

``` public interface CycleComputer ```

This interface is used to represent algorithms for identifying steady state
cycles (Type III extreme pathways) in stoichiometric networks.

**Author:**
:   This class was written and documented by Jeremiah Wright
    while in the Wagner lab.

---

| **Method Summary** | |
| --- | --- |
| `SparseDoubleMatrix<?,java.lang.String>` | `getCycles(IrrevRxnFluxome fluxome)`             Returns the set of all steady state cycles contained in the provided fluxome. |
| `ProgressMonitor` | `getProgress()`             Returns a progress monitor for this cycle computer. |

**Method Detail**

### getProgress

```
ProgressMonitor getProgress()
```

:   Returns a progress monitor for this cycle computer.

    :   **Returns:**: a progress monitor for this cycle computer.

---


### getCycles

```
SparseDoubleMatrix<?,java.lang.String> getCycles(IrrevRxnFluxome fluxome)
```

:   Returns the set of all steady state cycles contained in the provided
    fluxome.

    :   **Parameters:**: `fluxome` - the fluxome for which all cycles will be returned. **Returns:**: the extreme current matrix. Column variables are reaction names, and each row represents a steady state cycle.


---


|  |  |  |
| --- | --- | --- |
| **Overview** \| **Package** \| **Class** \| **Use** \| **Tree** \| **Deprecated** \| **Index** \| **Help** | |  |
| **PREV CLASS**   **NEXT CLASS** | **FRAMES**    **NO FRAMES**     **All Classes** |  |
| SUMMARY: NESTED \| FIELD \| CONSTR \| METHOD | DETAIL: FIELD \| CONSTR \| METHOD |  |


---
